# Prognostic significance of FT3 levels in hypertrophic cardiomyopathy patients with HFpEF

**DOI:** 10.3389/fcvm.2025.1546309

**Published:** 2025-08-21

**Authors:** Zhen-Yu Yang, Zhi-Yuan Zhang, Wei-Jie Zhang, Da Liu, Fu-Shi Piao, Xiangyu Yan, Fang-Tao Shi, Min Yang, Jing Chen, Sheng-song Zhu, Hong-Hou He, Pei-Hong Wu, Cheng Qian, Min Lin, Jun-Qing Feng, Chun-jin Lai

**Affiliations:** ^1^ Cardiac Surgery Department, The Peoplès Hospital of Anyang City, Anyang, Henan, China; ^2^ Department of Plastic and Burn Surgery, National Key Clinical Construction Specialty, The Affiliated Hospital of Southwest Medical University, Luzhou, Sichuan, China; ^3^ Department of Cardiology, the First Hospital of Hebei Medical University, Graduate School of Hebei Medical University, Shijiazhuang, Hebei, China; ^4^ Shenzhen Third People's Hospital, Shenzhen, China; ^5^ Department of Cardiology, Peking University Shenzhen Hospital, Shenzhen, China; ^6^ Institute of Disaster and Emergency Medicine, Tianjin University, Tianjin, China; ^7^ Department of Cardiology, State Key Laboratory of Cardiovascular Disease, Fuwai Hospital, National Center for Cardiovascular Diseases, Chinese Academy of Medical Sciences and Peking Union Medical College, Beijing, China; ^8^ Department of Cardiology and Institute of Vascular Medicine, Peking University Third Hospital, Beijing, China; ^9^ Division of Cardiac Arrhythmia, Cardiac and Vascular Center, The University of Hong Kong-Shenzhen Hospital, Shenzhen, Guangdong, China; ^10^ Department of Cardiology, Dongguan Tai-xin Hospital, Dongguan, China

**Keywords:** HCM-HFpEF, prognosis, FT3, thyroid function, all-cause mortality, cardiac transplantation, non-linear analysis

## Abstract

**Objective:**

This study sought to identify key prognostic factors in patients with hypertrophic cardiomyopathy (HCM) and heart failure with preserved ejection fraction (HFpEF), emphasizing the prognostic role of free triiodothyronine (FT3) levels.

**Research design and methods:**

This retrospective cohort study enrolled 992 HCM-HFpEF patients from two Chinese medical centers between 2009 and 2019, excluding those with thyroid-affecting medications or disorders. Data on demographic and clinical variables, including FT3, were analyzed using univariate and multivariate Cox regression, Kaplan–Meier (KM) survival analysis, and restricted cubic spline (RCS) analysis to explore prognostic factors and FT3's nonlinear predictive value.

**Results:**

Age, atrial fibrillation, and NT-proBNP levels significantly influenced prognosis, while FT3 emerged as an independent predictor of all-cause mortality and cardiac transplantation (*P* < 0.001). Patients with lower FT3 exhibited poorer long-term outcomes, with RCS identifying a critical threshold of 2.885 pg/ml (*P*-non-linear < 0.05).

**Conclusions:**

FT3 is a robust prognostic marker in HCM-HFpEF patients, supporting its integration into risk stratification. Clinicians should monitor FT3 levels, though intervention efficacy requires further prospective validation.

## Graphical Abstract

**Figure:**
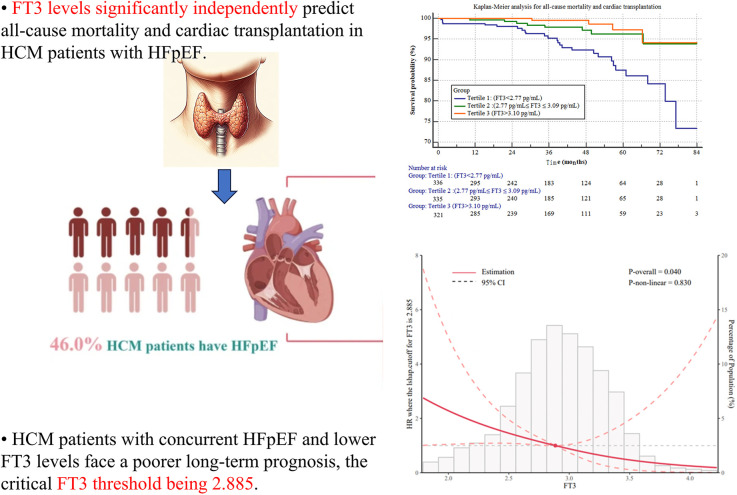

## Highlights

• FT3 independently predicts all-cause mortality and cardiac transplantation in HCM-HFpEF patients (*P* < 0.001).
• Lower FT3 levels in HCM-HFpEF patients are linked to worse long-term prognosis, with a critical threshold of 2.885 pg/ml identified via nonlinear RCS analysis (*P*-non-linear < 0.05).
• Age, atrial fibrillation, and NT-proBNP are significant prognostic factors in HCM-HFpEF, complementing FT3's role.
• Routine FT3 monitoring is recommended for risk stratification in HCM-HFpEF, though therapeutic interventions require further validation.

## 1 Introduction

Heart Failure (HF) is a chronic, progressive condition in which the heart cannot pump enough blood to meet the body's needs. HF remains a leading cause of global morbidity and mortality, affecting approximately 1%–2% of the population worldwide. The prognosis of HF varies widely, underscoring the critical need to identify prognostic factors to guide clinical decisions and enhance patient outcomes. Identifying these factors is vital for stratifying patients by risk of adverse events, tailoring individualized treatment plans, and tracking disease progression over time. Despite extensive research, novel prognostic markers are still required to refine risk prediction and optimize therapeutic strategies.

Heart Failure with Preserved Ejection Fraction (HFpEF) represents a distinct HF subtype characterized by a normal or near-normal ejection fraction, the proportion of blood ejected per heartbeat. Patients with HFpEF typically exhibit symptoms including dyspnea, fatigue, and exercise intolerance. The prevalence of HFpEF is rising, driven by an aging population and the increasing burden of HFpEF comorbidities such as hypertension and diabetes.

Hypertrophic Cardiomyopathy (HCM) is a genetic disorder marked by abnormal myocardial thickening, frequently resulting in arrhythmias, heart failure, or sudden cardiac death. Research indicates a high prevalence of HFpEF among HCM patients, with HFpEF established as an independent predictor of adverse outcomes. Heart failure, a frequent complication in both obstructive and non-obstructive. HCM, is independently associated with elevated mortality risk in this population (1). In HCM, heart failure predominantly manifests as HFpEF, driven by compromised ventricular diastolic function. Ventricular diastole encompasses active relaxation and passive filling phases: active relaxation depends on intrinsic myocardial properties, whereas passive filling is influenced by myocardial stiffness or compliance. HCM patients exhibit impairments in both active relaxation and passive filling. Approximately 46% of HCM patients develop HFpEF, substantially increasing the risk of end-stage heart failure and major adverse cardiac events (MACEs) (1, 2).

Thyroid hormones play a pivotal role in cardiovascular health, modulating heart rate, contractility, and vascular tone. The thyroid hormone profile serves as a key regulator of cardiovascular homeostasis under both physiological and pathological conditions (3, 4). Dysregulated thyroid hormone levels, such as those observed in hypothyroidism or hyperthyroidism, are linked to a spectrum of cardiovascular diseases, including heart failure (HF). Previous studies, including our own, supported by clinical evidence, have demonstrated that thyroid hormone levels, particularly free triiodothyronine (FT3), correlate with deteriorating cardiac function (5–8). Moreover, hypothyroidism has been identified as an independent risk factor for adverse cardiovascular events. The impact of thyroid hormones on cardiovascular disease may arise from their regulation of gene expression and signaling pathways tied to cardiac hypertrophy, fibrosis, and inflammation (9, 10). FT3, a principal thyroid hormone, is vital for maintaining cardiac function. Existing research suggests that reduced FT3 levels are associated with poorer outcomes in HF patients (10). However, the prognostic role of FT3 in HCM patients with HFpEF remains poorly defined, despite prior investigations into FT3 in broader HCM cohorts (7). Further research is needed to elucidate this relationship and establish optimal FT3 cutoff values for risk stratification.

Given the elevated prevalence of HFpEF in HCM patients and the established influence of thyroid hormones on cardiovascular pathology, identifying prognostic markers for this population is imperative. This study seeks to assess the prognostic significance of FT3 levels in HCM patients with HFpEF, utilizing a larger cohort and advanced statistical modeling to address this gap, while also exploring potential underlying mechanisms.

## 2 Research design and methods

### 2.1 Ethics statement

This study adhered to the ethical principles outlined in the Declaration of Helsinki and complied with China's regulations and guidelines on good clinical practice. It was approved by the Ethics Committee of The People's Hospital of Anyang City. Written informed consent was obtained from all participants prior to study initiation.

### 2.2 Design, patients, and outcome measure

#### 2.2.1 Study patients

This retrospective cohort study was conducted across two medical centers: The People's Hospital of Anyang City and Fuwai Hospital (National Center for Cardiovascular Diseases, China). Between October 1, 2009, and December 31, 2019, a total of 2,802 patients (aged ≥16 years) were diagnosed with hypertrophic obstructive cardiomyopathy (HOCM). Of these, 187 patients with EF <50% and patients using medications known to affect thyroid function (e.g., thyroxine, liothyronine, amiodarone, corticosteroids, or antithyroid drugs) or those with a documented history of thyroid disorders (e.g., hyperthyroidism or hypothyroidism), hypothalamic-pituitary diseases, or autoimmune conditions were excluded. After applying these criteria, 992 patients with hypertrophic cardiomyopathy and HFpEF were enrolled. Patients were stratified into three tertiles based on FT3 levels: Tertile 1 (FT3 <2.77 pg/ml, *n* = 336), Tertile 2 (2.77 pg/ml ≤FT3 ≤3.09 pg/ml, *n* = 335), and Tertile 3 (FT3 >3.10 pg/ml, *n* = 321).

#### 2.2.2 Diagnosis of HCM

The diagnosis of HCM was based on established criteria (11–13): (1) wall thickness ≥15 mm in one or more left ventricular (LV) myocardial segments as assessed by echocardiography, cardiac magnetic resonance imaging, or computed tomography; (2) wall thickness of 13–14 mm along with additional findings such as a family history, non-cardiac symptoms and signs, electrocardiogram (ECG) abnormalities, laboratory tests, and multi-modality cardiac imaging; (3) presence of left ventricular outflow tract obstruction (LVOTO), characterized by dynamic LVOT obstruction caused by systolic anterior motion of the mitral valve, with an LVOT gradient ≥30 mmHg at rest or during physiological provocations (e.g., Valsalva maneuver, standing, or exercise). Significant dynamic LVOT obstruction was confirmed using 2-dimensional and Doppler echocardiography. In cases where echocardiography was inconclusive, invasive hemodynamic catheterization with provocation was performed.

#### 2.2.3 Diagnosis of HFpEF

HFpEF diagnosis in this study was established by assessing a combination of symptoms, signs, left ventricular ejection fraction (LVEF), and NT-proBNP levels. We adhered to the 2017 American Heart Association (AHA) criteria (1, 14), defining HFpEF as LVEF ≥50%, NT-proBNP ≥800 pg/ml, and the presence of heart failure symptoms or signs. Evidence of left ventricular diastolic dysfunction was required, confirmed via echocardiography using parameters indicative of impaired LV relaxation, filling, or elevated left atrial pressure. These included an elevated E/e' ratio (>9), increased left atrial volume index, and markers of LV relaxation abnormalities (e.g., prolonged mitral valve deceleration time). Patients not fulfilling these criteria were classified as non-HF.

#### 2.2.4 Thyroid function test

Thyroid function was evaluated after stabilization of heart failure symptoms with standard oral medications prior to discharge, avoiding the acute phase to minimize confounding by non-thyroidal illness. Tests were conducted at a median of 10 days post-admission. Blood samples were collected following a 12-h fast, and serum levels of thyroid hormones (including FT3, FT4) and thyroid-stimulating hormone (TSH) were quantified using radioimmunoassay (Immulite 2000; Siemens, Germany).

### 2.3 Follow-up and endpoints

Follow-up began at the patient's first clinic visit after October 1, 2009. At baseline, patients were evaluated for age, sex, NYHA functional class, maximum left ventricular wall thickness (LVWT), maximum LVOT gradient (provocable), left ventricular function, atrial fibrillation, and conventional risk factors for cardiac death.

The primary endpoints were all-cause mortality and cardiac transplantation during long-term follow-up. Data on mortality and adverse events were obtained from hospital records, civil service population registers, and information provided by patients or their general practitioners. Patients lost to follow-up were censored at their last point of contact. If no events occurred during follow-up, patients were administratively censored on December 31, 2019. The primary endpoint was a composite of all-cause mortality and heart transplantation, reflecting severe disease progression in HFpEF. To ensure the robustness of the results, sensitivity analyses were conducted separately for all-cause mortality and heart transplantation.

### 2.4 Data analysis

Statistical analyses were performed using SPSS version 27.0 for Windows and R software version 4.2.2 (R Foundation for Statistical Computing). Continuous variables are presented as means ± SD, and analysis of variance (ANOVA) was used to compare means across groups. Relationships between parametric variables were analyzed using multiple linear regression. Baseline differences between treatment groups were evaluated using bivariable analyses, including *χ*^2^ tests, Fisher's exact test, Student's *t*-test, and Kruskal–Wallis ANOVA. The Cox proportional hazards model was used to estimate hazard ratios (HRs). Kaplan–Meier analysis was applied to evaluate cumulative survival across different groups. The variables analyzed (including age, gender, NT-proBNP, atrial fibrillation, etc.) were selected based on their known associations with prognosis in HFpEF and HCM, as supported by previous studies (15–20). A *P*-value < 0.05 was considered statistically significant.

Restricted cubic splines (RCS) were utilized to model nonlinear associations between continuous predictors and outcomes, extending linear regression with cubic polynomial functions for flexible relationship modeling. In this study, RCS effectively captured the nonlinear association between FT3 levels and cardiovascular events in R. Four knots were placed at the 10th, 50th, and 90th percentiles of the FT3 distribution to balance flexibility and parsimony. The critical FT3 threshold of 2.885 pg/ml was derived from the RCS curve, identifying the inflection point where event risk markedly increased. Model fit was assessed using the likelihood ratio test, with spline shapes visually inspected via graphical displays.

Initially, an exploratory analysis was conducted by dividing patients into tertiles based on FT3 levels. Subsequently, using the RCS-derived threshold of 2.885 pg/ml, patients were categorized into two groups (FT3 ≤2.885 pg/ml and FT3 >2.885 pg/ml) for confirmatory subgroup analysis.

### 2.5 Data and resource availability statement

Datasets generated and analyzed in this study are available from the corresponding author upon reasonable request, subject to ethical and legal constraints.

## 3 Results

The study included 992 HCM patients with HFpEF, with a mean age of 53.60 ± 13.83 years. Most patients were male (58.2%). Atrial fibrillation was observed in 22.5% of patients, and 21.0% had unexplained syncope. The mean NT-proBNP level was 2,278.18 ± 1,743.44 fmol/ml, and the mean FT3 level was 2.94 ± 0.58 pg/ml.

Table 1 summarizes the baseline clinical characteristics of the study population, stratified into three tertiles based on FT3 levels. The study population consisted of 992 participants: 336 in Tertile 1, 335 in Tertile 2, and 321 in Tertile 3. The mean age of the total population was 53.60 ± 13.83 years. Tertile 1 had the oldest participants (57.21 ± 13.84 years), while Tertile 3 had the youngest (50.00 ± 13.49 years) (*p* < 0.001). Male participants accounted for 58.2% of the population, with Tertile 3 showing the highest proportion of males (76.0%) (*p* < 0.001). Body mass index (BMI), systolic and diastolic blood pressures, and left ventricular (LV) end-diastolic diameter exhibited no significant inter-tertile differences. Participants in Tertile 3 had significantly higher FT3 levels compared to those in Tertile 1 and Tertile 2 (*p* < 0.001). Atrial fibrillation was more common in Tertile 3 than in Tertile 1 or Tertile 2 (*p* < 0.001). NYHA Class III or IV was more frequent in Tertile 1 compared to Tertile 2 and Tertile 3 (*p* < 0.001). The use of beta-blockers, ACEI/ARBs, diuretics, and calcium channel blockers did not differ significantly among tertiles.

**Table 1:** Baseline clinical characteristics of the study population divided into the three tertiles based on the level of FT3.

Variables	Total (*n* = 992)	Tertile 1 (*n* = 336)	Tertile 2 (*n* = 335)	Tertile 3 (*n* = 321)	*P*-value
Age, y	53.60 ± 13.83	57.21 ± 13.84	53.60 ± 13.30	50.00 ± 13.49	<0.001
Male, *n* (%)	577 (58.2%)	132 (39.3%)	201 (60.0%)	244 (76.0%)	<0.001
BMI (kg/m^2^)	25.42 ± 4.25	25.01 ± 3.59	25.51 ± 4.92	25.72 ± 4.07	0.177
Systolic BP (mmHg)	121.58 ± 18.47	122.17 ± 18.06	120.84 ± 19.27	121.79 ± 18.05	0.716
Diastolic BP (mmHg)	74.12 ± 12.27	73.97 ± 11.98	74.01 ± 13.15	74.37 ± 11.59	0.927
NT-proBNP (fmol/ml)	2,278.18 ± 1,743.44	2,760.72 ± 2,024.43	2,153.81 ± 1,610.95	1,930.14 ± 1,454.51	<0.001
FT3 (pg/ml)	2.94 ± 0.58	2.456 ± 0.29	2.93 ± 0.09	3.44 ± 0.69	<0.001
Atrial fibrillation, *n* (%)	223 (22.5%)	55 (16.4%)	21 (6.2%)	31 (9.7%)	<0.001
Non- sustained ventricular tachycardia, *n* (%)	52 (5.2%)	19 (5.7%)	15 (4.5%)	18 (5.6%)	0.742
NYHA Class Ⅲ or Ⅳ, *n* (%)	128 (12.9%)	63 (18.8%)	35 (10.4%)	30 (9.4%)	<0.001
Unexplained syncope, *n* (%)	208 (21.0%)	57 (17.0%)	91 (27.2%)	60 (18.7%)	0.002
Echocardiography					
Interventricular septal thickness (mm)	20.23 ± 5.19	19.51 ± 4.86	20.57 ± 5.68	20.56 ± 4.90	0.038
LV end-diastolic diameter (mm)	42.92 ± 5.91	42.86 ± 5.80	42.64 ± 6.33	43.30 ± 5.53	0.464
LV ejection fraction (%)	64.55 ± 6.10	63.97 ± 6.58	64.38 ± 6.00	65.30 ± 5.65	0.054
LV outflow tract gradient, at rest (mmHg)	51.00 ± 13.42	46.17 ± 15.48	52.73 ± 12.59	53.96 ± 12.29	0.090
Medications					
Beta-blocker, *n* (%)	603 (60.8%)	205 (61.0%)	199 (59.4%)	199 (61.9%)	0.465
ACEI/ARB, *n* (%)	170 (17.1%)	63 (18.8%)	60 (17.9%)	47 (14.6%)	0.449
Diuretic, *n* (%)	148 (19.0%)	72 (21.4%)	55 (16.4%)	61 (19.0%)	0.120
Calcium antagonist, *n* (%)	205 (20.7%)	84 (25.0%)	58 (17.3%)	63 (19.6%)	0.091

Values are mean ± SD or *n* (%).

Abbreviation: BMI, body mass index; NT-proBNP, N-terminal pro-brain natriuretic peptide; BP, blood pressure; NYHA, New York Heart Association; LV, left ventricle; ACEI/ARB, angiotensin-converting enzyme inhibitor/angiotensin receptor blocker.

Table 2 presents the results of the univariate Cox analysis for all-cause mortality and cardiac transplantation. Hazard ratios (HR) and 95% confidence intervals (CI) are reported for each variable, along with their corresponding *p*-values. Higher FT3 levels are significantly associated with a lower risk of all-cause mortality and cardiac transplantation (HR: 0.206, 95% CI: 0.106–0.402, *p* < 0.001). Older age is associated with an increased risk of mortality and transplantation (HR: 1.085, 95% CI: 1.053–1.118, *p* < 0.001). Male gender is not significantly associated with mortality or transplantation risk (HR: 0.593, 95% CI: 0.314–1.122, *p* = 0.108). Elevated NT-proBNP levels are associated with an increased risk of mortality and transplantation (HR: 1.026, 95% CI: 1.015–1.037, *p* < 0.001). Atrial fibrillation history is significantly associated with a higher risk of mortality and transplantation (HR: 4.240, 95% CI: 2.235–8.044, *p* < 0.001). Unexplained syncope history is not significantly associated with mortality or transplantation risk (HR: 1.460, 95% CI: 0.724–2.944, *p* = 0.287). Interventricular septal thickness is not significantly associated with mortality or transplantation risk (HR: 1.001, 95% CI: 0.948–1.056, *p* = 0.979).

**Table 2:** Univariate cox analysis for all-cause mortality and cardiac transplantation.

Variables	HR (95% CI)	*P* value
FT3 (pg/ml)	0.206 (0.106, 0.402)	<0.001
Age, y	1.085 (1.053, 1.118)	<0.001
Male, *n* (%)	0.593 (0.314, 1.122)	0.108
NT-proBNP (per 100 fmol/ml)	1.026 (1.015, 1.037)	<0.001
Atrial fibrillation, *n* (%)	4.240 (2.235, 8.044)	<0.001
Unexplained syncope, *n* (%)	1.460 (0.724, 2.944)	0.290
Interventricular septal thickness (mm)	1.001 (0.948, 1.056)	0.980

Abbreviations are listed in Table 1.

Table 3 presents two multivariate Cox regression models: Model 1 and Model 2. Both models examine the relationship between various factors and all-cause mortality and cardiac transplantation. Model 1 includes age, atrial fibrillation, NT-proBNP (per 100 fmol/ml), and FT3 (pg/ml). The hazard ratio (HR) for age was 1.062 (95% CI: 1.029–1.096, *p* < 0.001), indicating that each 1-year increase in age raised the risk of all-cause mortality and cardiac transplantation by 6.2%. The HR for atrial fibrillation was 2.142 (95% CI: 1.084–4.234, *P* = 0.028), indicating that atrial fibrillation was associated with a 2.1-fold higher risk of all-cause mortality and cardiac transplantation. The HR for NT-proBNP was 1.021 (95% CI: 1.007–1.035, *P* = 0.003), indicating that each 100 fmol/ml increase in NT-proBNP raised the risk of all-cause mortality and cardiac transplantation by 2.1%. The HR for FT3 was 0.374 (95% CI: 0.178–0.788, *P* = 0.010), indicating that each 1 pg/ml increase in FT3 reduced the risk of all-cause mortality and cardiac transplantation by 62.6%. Model 2 included the same factors as Model 1, but FT3 was analyzed categorically (tertiles). The reference group was FT3 Tertile 3, and the HR for FT3 Tertile 2 was 0.480 (95% CI: 0.214–1.074, *P* = 0.074), indicating a 52.0% lower risk of all-cause mortality and cardiac transplantation compared to the reference group. The HR for FT3 Tertile 1 was 0.336 (95% CI: 0.114–0.988, *P* = 0.048), indicating a 66.4% lower risk of all-cause mortality and cardiac transplantation compared to the reference group. Additionally, age, atrial fibrillation, and NT-proBNP remained significant in Model 2. The HR for age was 1.040 (95% CI: 1.002–1.079, *P* = 0.037), indicating that each one-year increase in age raised the risk of all-cause mortality and cardiac transplantation by 4.0%. The HR for atrial fibrillation was 3.399 (95% CI: 1.356–8.517, *P* = 0.009), indicating that atrial fibrillation was associated with a 3.4-fold higher risk of all-cause mortality and cardiac transplantation. The HR for NT-proBNP was 1.023 (95% CI: 1.005–1.041, *P* = 0.012), indicating that each 100 fmol/ml increase in NT-proBNP raised the risk of all-cause mortality and cardiac transplantation by 2.3%.

**Table 3:** Multivariate cox analysis for all-cause mortality and cardiac transplantation.

Variables	*n* (Events)	Model 1		Model 2	
		HR (95% CI)	*P* value	HR (95% CI)	*P* value
Age, y	--	1.062 (1.029, 1.096)	<0.001	1.040 (1.002, 1.079)	0.037
Atrial fibrillation	--	2.142 (1.084, 4.234)	0.028	3.399 (1.356, 8.517)	0.009
NT-proBNP (per 100 fmol/ml)	--	1.021 (1.007, 1.035)	0.003	1.023 (1.005,1.041)	0.012
Male, n (%)	--	0.700 (0.360, 1.350)	0.285	0.720 (0.375, 1.380)	0.325
FT3 ((pg/mL)	--	0.374 (0.178, 0.788)	0.010	--	--
FT3 Tertial 3	321 (40)	--	--	1 (reference)	--
FT3 Tertial 2	335 (45)	--	--	0.480 (0.214, 1.074)	0.074
FT3 Tertial 1	336 (50)	--	--	0.336 (0.114, 0.988)	0.048

Notes: Model 1 and Model 2 are adjusted for age, gender, NT-proBNP, atrial fibrillation, unexplained syncope, and interventricular septal thickness.

*n* (Events): Represents the total number of patients and the number of outcome events in each FT3 tertile group. For example, in FT3 Tertile 3, there are 321 patients, of whom 40 experienced the outcome event.

**Figure 1:**
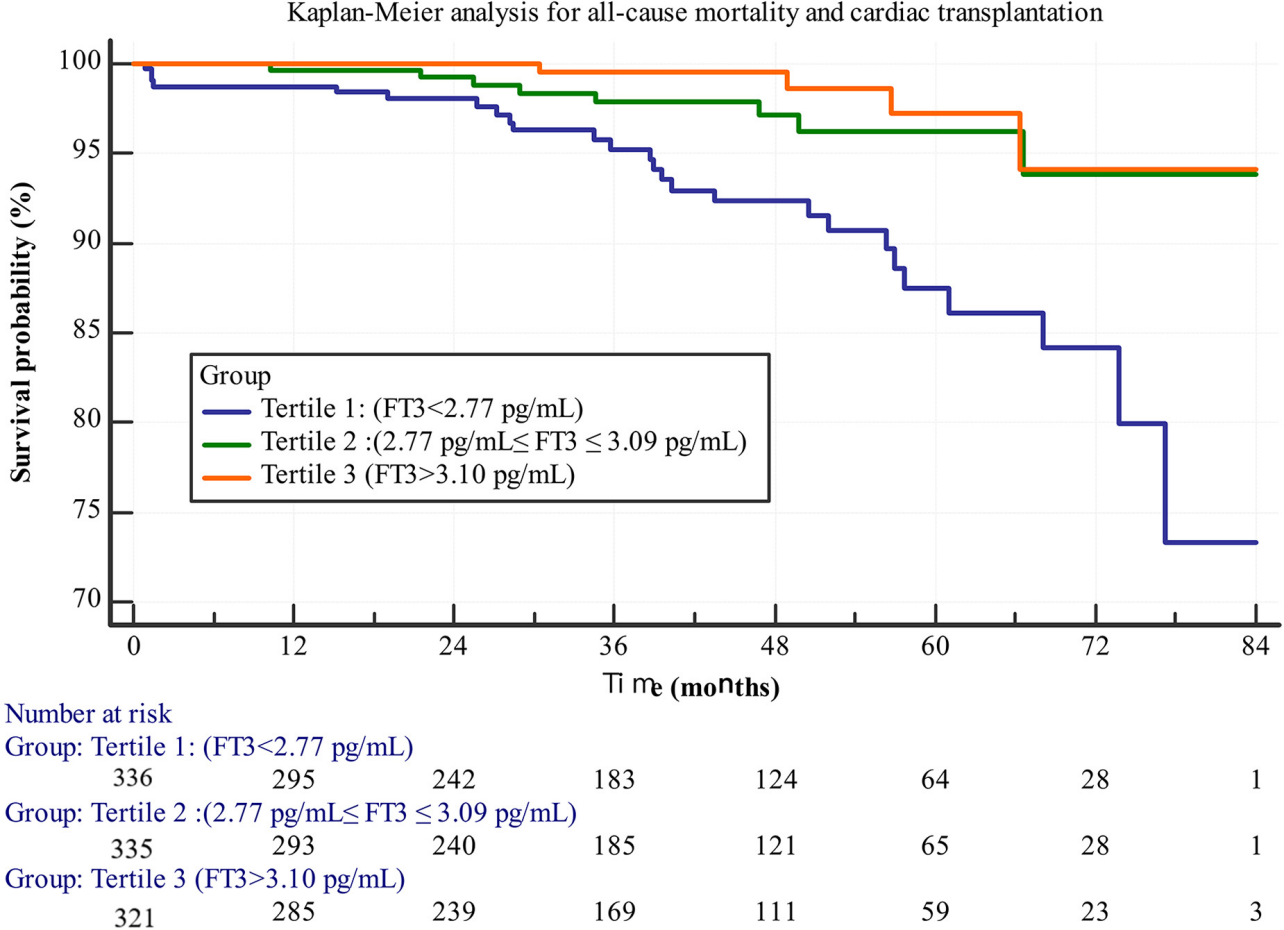
Kaplan-Meier survival curves for all-cause mortality and cardiac transplantation stratified by FT3 tertiles.

Figure 1 shows the Kaplan–Meier (KM) survival curve, indicating that Group 1 had the poorest long-term prognosis. A statistically significant difference was observed among the three groups (log-rank test, *p* < 0.001). The KM survival curve graphically represents survival probabilities over time and facilitates comparisons between different groups. In this study, patients with lower FT3 levels exhibited worse prognoses compared to those with higher FT3 levels.

**Figure 2:**
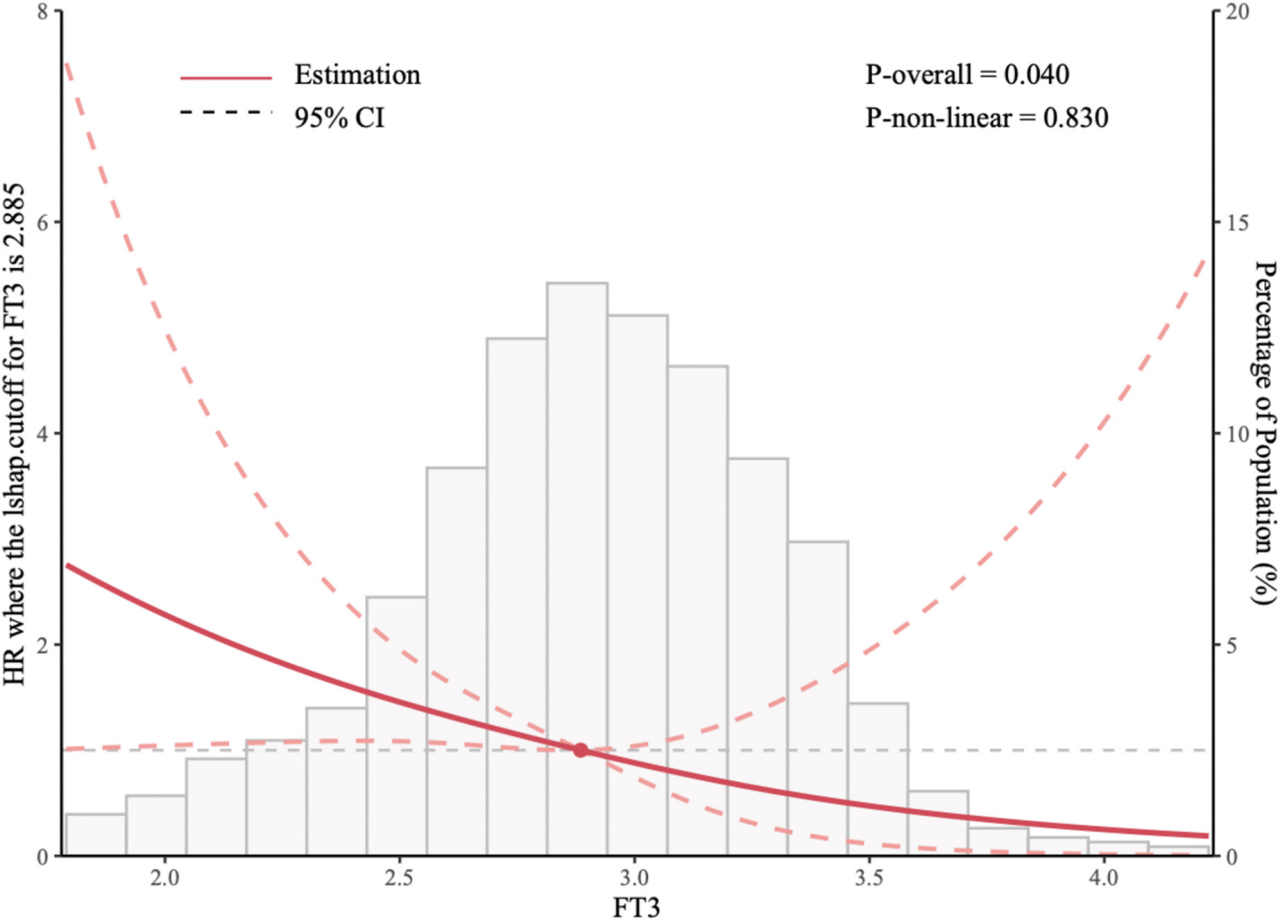
Restricted cubic splines (RCS) of the relationship between the FT3 and the risk of all-cause mortality and cardiac transplantation.

Figure 2 employs restricted cubic splines (RCS) to model the nonlinear association between FT3 levels and the risk of all-cause mortality and cardiac transplantation. Four knots were positioned at the 10th, 50th, and 90th percentiles of the FT3 distribution to facilitate flexible yet parsimonious modeling of this relationship. Model fit was evaluated using the likelihood ratio test, and the spline shape was inspected via graphical displays. The RCS analysis confirmed a nonlinear relationship, with an “L-shaped” curve indicating that lower FT3 levels correlated with progressively worse outcomes. The critical FT3 threshold of 2.885 pg/ml, identified as the inflection point where risk sharply increased, was derived from this analysis.

Subgroup analysis using the 2.885 pg/ml threshold further confirmed that the survival rate was significantly lower in the FT3 ≤2.885 pg/ml group (log-rank *P* < 0.001). In sensitivity analyses, multivariable Cox regression models conducted separately for all-cause mortality and heart transplantation demonstrated that lower FT3 levels were significantly associated with an increased risk for both endpoints. Specifically, FT3 (as a continuous variable) was associated with all-cause mortality with an HR of 0.40 (95% CI: 0.20–0.80, *P* = 0.009) and with heart transplantation with an HR of 0.35 (95% CI: 0.15–0.82, *P* = 0.015). These findings indicate a consistent inverse relationship between FT3 and each component of the composite endpoint, confirming the robustness of the composite endpoint selection.

## 4 Discussion

This study assessed the prognostic significance of free triiodothyronine (FT3) levels in patients with hypertrophic cardiomyopathy (HCM) and heart failure with preserved ejection fraction (HFpEF). Our results demonstrate that lower FT3 levels are independently associated with an elevated risk of all-cause mortality and cardiac transplantation, even after adjusting for confounders such as age, atrial fibrillation, and NT-proBNP in multivariate Cox models. Although gender was not significant in the multivariable model, it was included as a covariate due to its established role in HFpEF. Kaplan–Meier (KM) survival analysis confirmed poorer long-term outcomes in patients with lower FT3 levels, while restricted cubic spline (RCS) analysis revealed a nonlinear, “L-shaped” relationship between FT3 and prognosis, with a critical threshold of 2.885 pg/ml marking a sharp increase in risk. The initial tertile analysis provided an overall trend of FT3's impact, while the RCS analysis, by identifying a specific threshold, further refined the interpretation of the results. Due to challenges in accurately distinguishing causes of death in the dataset and to maximize statistical power, we chose all-cause mortality over cardiovascular death. However, heart transplantation may be influenced by resource availability, a limitation partially addressed by separate analyses.

Comparison with Previous Studies Our findings align with prior research linking low FT3 levels to adverse outcomes in heart failure patients (21, 22). Notably, studies like Zhang et al. (7) have explored FT3's prognostic role in hypertrophic obstructive cardiomyopathy, yet its specific significance in the HCM-HFpEF subgroup remained underexplored. Our study addresses this gap with a larger cohort (*n* = 992), comprehensive adjustment for clinical covariates, and the application of RCS analysis to delineate a nonlinear FT3-prognosis relationship, offering a novel contribution to the field. Interpretation of Results These results underscore FT3 as a robust prognostic marker in HCM-HFpEF patients. Lower FT3 levels correlate with worse outcomes, enabling identification of high-risk individuals who may benefit from intensified monitoring or tailored interventions. The RCS analysis, with a significant nonlinear *P*-value (<0.05), revealed an “L-shaped” curve, indicating that risk escalates sharply below a critical FT3 threshold of 2.885 pg/ml, beyond which further decreases yield diminishing additional impact.

Clinical Implications Identifying FT3 as an independent prognostic factor holds substantial implications for managing HCM-HFpEF patients. Clinicians should routinely assess FT3 levels to stratify risk and guide monitoring strategies. While our data suggest that low FT3 is tied to poorer outcomes, direct evidence for FT3 optimization (e.g., thyroid hormone supplementation) remains limited. Prospective trials are needed to establish intervention thresholds, such as FT3 <2.885 pg/ml, and evaluate the efficacy of thyroid function-targeted therapies in improving prognosis. Thyroid Hormones and Cardiovascular Function Mounting evidence underscores thyroid hormones as pivotal regulators of cardiovascular physiology. Thyroid hormone deficiency (hypothyroidism) is implicated in various cardiovascular pathologies, including heart failure, while excess (hyperthyroidism) is associated with adverse events like atrial fibrillation and sudden cardiac death. In this study, FT3's prognostic role may reflect its direct influence on cardiac function or serve as a marker of disease severity, such as non-thyroidal illness syndrome (NTIS) in advanced HFpEF. Mechanistic Insights The mechanisms linking thyroid hormones to cardiovascular outcomes are multifaceted and incompletely elucidated. Thyroid hormones modulate nearly all facets of cardiac function, including contractility, heart rate, rhythm, and the structure and function of blood vessels (23, 24). Additionally, thyroid hormones exert significant effects on lipid and carbohydrate metabolism, further influencing cardiovascular health (25).

In HCM-HFpEF, low FT3 may exacerbate diastolic dysfunction by impairing myocardial relaxation, a hypothesis warranting further exploration. Potential Mechanisms One mechanism by which thyroid hormones may affect HFpEF prognosis is through their modulation of the sympathetic nervous system (26). By enhancing sympathetic activity, 284 thyroid hormones elevate heart rate and blood pressure, potentially driving cardiac hypertrophy and 285 remodeling processes that could accelerate heart failure progression (27).

In severe HFpEF, reduced 286 peripheral conversion of T4 to T3 (e.g., via NTIS) may further lower FT3 levels, compounding these effects. Another plausible mechanism involves thyroid hormone interactions with the renin-angiotensin aldosterone system (RAAS), a critical regulator of blood pressure and fluid homeostasis (26). Thyroid hormones activate RAAS, promoting sodium and water retention, which may worsen HFpEF symptoms, particularly in patients with low FT3.

### 4.1 Limitations of the study

Limitations This retrospective study has several limitations. Its design introduces potential selection bias from non-random patient inclusion and residual confounding from unmeasured variables (e.g., lifestyle or environmental factors). Conducted across two Chinese centers, the findings may not fully generalize to other populations due to regional differences in healthcare practices and patient demographics. Ethnic and genetic variations in thyroid hormone metabolism (e.g., deiodinase activity) or HCM pathology (e.g., MYH7 mutations) could also influence FT3's prognostic role, limiting broader applicability. Future Directions Future research should elucidate the mechanisms linking FT3 to HFpEF prognosis, including the potential role of NTIS and the FT3/FT4 ratio as markers of peripheral thyroid metabolism (e.g., PMID: 37285810, PMID: 35498022). Prospective, multicenter studies with diverse, larger cohorts are needed to validate our findings, refine the optimal FT3 threshold (e.g., via ROC analysis), and assess generalizability across ethnic groups. Interventional trials are also critical to determine whether optimizing FT3 levels improves outcomes. Clinical Recommendations Our findings advocate for routine FT3 assessment in HCM-HFpEF patients, alongside established markers like age, atrial fibrillation, and NT-proBNP, to enhance risk stratification. Patients with low FT3 (<2.885 pg/ml) warrant closer monitoring for adverse events. While targeted interventions to improve thyroid function (e.g., addressing hypothyroidism or NTIS) may hold promise, their efficacy remains unproven, necessitating further study. This study highlights the value of addressing modifiable factors like thyroid dysfunction in HFpEF management.

## 5 Conclusion

This study establishes FT3 as an independent prognostic factor in HCM-HFpEF patients, with a nonlinear, “L-shaped” relationship to outcomes (*P*-non-linear $<0.05$) and a critical threshold of 2.885 pg/ml. These insights are pivotal for clinicians to identify high-risk patients for tailored management. Routine FT3 monitoring could facilitate early intervention, though its therapeutic modulation requires further validation. Future studies should explore FT3's mechanistic links to cardiovascular events, including NTIS and FT3/FT4 dynamics, and evaluate intervention efficacy across diverse populations.

However, our study is constrained by limitations such as its retrospective design, potential unmeasured confounders (e.g., lifestyle factors), and restricted generalizability due to its focus on two Chinese centers. Larger, prospective studies with comprehensive datasets and multi-ethnic cohorts are essential to confirm these findings, refine the FT3 threshold, and clarify underlying mechanisms. Collectively, this work highlights FT3 as a key prognostic marker in HCM-HFpEF patients, providing novel insights for clinical management.

## Data Availability

The datasets presented in this article are not readily available because the dataset is available upon reasonable request to the corresponding author, subject to ethical and legal requirements for data sharing, including ensuring no participant-identifiable information is disclosed. Requests to access the datasets should be directed to Chunjin Lai, 2111110456@alumni.pku.edu.cn.
